# The Significance of Lower Limb Asymmetry in the Assessment of Spinal Deformities in Individuals with Scoliotic Posture and Scoliosis

**DOI:** 10.3390/jcm15208065

**Published:** 2026-10-17

**Authors:** Anna Puchalska-Sarna, Katarzyna Walicka-Cupryś, Jadwiga Trzeciak, Ewa Lenart-Domka, Rafał Baran, Paweł Guz, Anna Kinasz-Jakubiec, Joanna Baran

**Affiliations:** 1 University of Rzeszów, Faculty of Health Sciences and Psychology, Institute of Physiotherapy, Al. T. Rejtana 16C, 35-959 Rzeszów, Poland; apuchalska@ur.edu.pl (A.P.-S.); kwalicka@ur.edu.pl (K.W.-C.); rbaran@ur.edu.pl (R.B.); 2 University Centre for Research and Development in Health Sciences, University of Rzeszów, Al T. Rejtana 16C, 35-959 Rzeszów, Poland; 3 St. Queen Jadwiga Provincial Clinical Hospital in Rzeszów, ul. Lwowska 60, 35-301 Rzeszów, Poland; jtrzeciak@ur.edu.pl (J.T.); edomka565@gmail.com (E.L.-D.); pguz.pl@gmail.com (P.G.); kinasza@poczta.onet.pl (A.K.-J.); 4 Faculty of Medical Sciences, University of Rzeszów, ul. Al. T. Rejtana 16C, 35-959 Rzeszow, Poland

**Keywords:** scoliosis, leg length discrepancy, scoliometer, pelvic obliquity, clinical tests

## Abstract

**Background:** Adolescent Idiopathic Scoliosis (AIS) and scoliotic posture present complex three-dimensional interactions between pelvic obliquity and spinal alignment. Leg length discrepancy (LLD) is frequently observed in individuals presenting with scoliotic posture and is associated with pelvic obliquity and compensatory posture. This study evaluated the relationship between lower limb asymmetry and spinal curvature, incorporating age-restricted and curve-severity sensitivity analyses. **Methods:** Out of 65 individuals screened for scoliotic posture, 38 participants were included in the final analysis (mean age 15.3 years; 19 with structural scoliosis [Cobb ≥ 10°] and 19 with non-structural postural asymmetry). Clinical evaluation included the angle of trunk rotation (ATR) using Bunnell’s scoliometer, the long-sitting test (Derbolovsky test), the standing flexion test, and the Gillet test. Radiological assessment (X-ray) was used to measure the Cobb angle, vertebral rotation (Nash–Moe scale), and LLD. Statistical analysis was performed using Spearman’s rank correlation and comparative tests (Student’s *t*-test, Welch’s test, and Mann–Whitney U test). Sensitivity analyses were conducted for pediatric participants aged < 18 years (*n* = 35) and structural scoliosis cases (Cobb ≥ 10°, *n* = 19) Bonferroni correction was applied for multiple testing ($p_{adj}$). **Results:** In the total cohort, ATR correlated moderately with radiographic LLD *(r_s_* = 0.40, *p_adj_* = 0.041) and pelvic height difference *(r_s_* = 0.40, *p_adj_* = 0.04). However, these primary associations attenuated and lost statistical significance in both the under-18 (*p_adj_* = 0.114 and *p_adj_* = 0.128) and structural scoliosis (*p_adj_* = 0.271 and *p_adj_* = 0.279) subgroups. Conversely, the association between ATR and absolute leg-length difference remained robust and statistically significant in the under-18 subgroup *(r_s_* = 0.50, *p_adj_* = 0.021). Lower limb asymmetry indicators showed no significant correlations with the Cobb angle across any analyses *(p_adj_* = 1.000). Clinical pelvic tests did not differentiate ATR or Cobb values, but left-sided positives were associated with significantly greater cervical lordosis *(p_adj_* < 0.025). **Conclusions:** Scoliometer measurements reflect surface trunk rotation rather than structural curve severity (Cobb angle). Primary LLD associations with ATR are sensitive to cohort maturity and curve structurality, losing significance in subgroup analyses. Only absolute leg-length difference maintained a robust association with ATR in the adolescent cohort. Clinical interpretation of scoliometer findings must account for lower limb posture while recognizing that surface asymmetry does not directly mirror structural scoliosis magnitude.

## 1. Introduction

Adolescent Idiopathic Scoliosis (AIS) is a three-dimensional deformity of the spine with a lateral curvature of the spine in the frontal plane, accompanied by vertebral rotation and abnormalities in the physiological curves in the sagittal plane [1]. Despite the advances in other imaging methods, a two-dimensional (2D) posteroanterior (PA) X-ray of the full-length spine is still considered the primary tool for the initial evaluation and long-term follow-up of AIS [2].

In daily clinical practice, standing PA X-rays often reveal leg length discrepancy (LLD) in patients with scoliotic posture or AIS. This discrepancy may be classified as functional if it results from body posture and mechanical abnormalities, or structural if it is a consequence of the anatomically shorter length of the bones [3]. Measuring the asymmetry in femoral head height on standing radiographs is a simple and well-established method for the functional assessment of LLD [4]. Furthermore, it has been pointed out that the use of three-dimensional (3D) imaging techniques in measuring the length of the lower limbs presents certain limitations [5].

LLD is a common condition in orthopaedic practice and is defined as a difference in the length of the right and left leg. It is usually mild—it is estimated that a difference of less than 10 mm is observed in approximately 90%, while complete symmetry in leg length is found in only about 10% of the general population [6].

The most common causes of LLD include growth disorders, excessive unilateral growth, and congenital or acquired dysplasia of the lower extremities [7]. A severe difference in leg length can lead to clinical consequences. Recent studies suggest there is a link between LLD and lower back pain, disc pathology, osteoarthritis of the hip and knee, stress fractures, and balance disorders while standing, as well as an increased risk of running injuries [8,9,10,11]. Although these complications are rarely observed in children and adolescents, they may develop later, particularly in adulthood.

The most common adaptations of the skeletal system in response to LLD include pelvic obliquity, pelvic rotation, and secondary curves of the spine [12]. Discrepancy in leg length causes the pelvis to be tilted, impairing the symmetry of the hip girdle. In order to maintain the horizontal alignment of the shoulder girdle, the body may compensate by developing a lateral curve of the spine. This type of curve, referred to as functional scoliosis, usually regresses completely or partially after LLD has been corrected [13]. When evaluating lower limb asymmetry, a clear clinical distinction must be made between anatomical (structural) leg length discrepancy (LLD), which stems from true osseous length differences in the femur or tibia, and functional (apparent) LLD, which arises from altered joint positioning, foot arch morphology, or pelvic obliquity without underlying bone shortening. Furthermore, two distinct clinical scenarios should be differentiated regarding spinal alignment: Primary LLD with secondary spinal adaptation means that an anatomical or functional LLD can be associated with compensatory pelvic obliquity, generating a functional lateral curvature of the spine (functional scoliosis) to maintain postural balance. Primary scoliosis with secondary apparent LLD means that, conversely, primary structural scoliosis and associated trunk asymmetry can alter pelvic orientation and weight distribution, resulting in a secondary, apparent (functional) LLD.

While prolonged asymmetric loading associated with functional scoliosis has been hypothesised to contribute to structural spinal remodelling over time, this potential progression remains controversial and is not fully supported by conclusive clinical evidence. Therefore, functional adaptations and true structural deformities must be evaluated with caution [14,15,16].

For this reason, early diagnosis and adequate procedures in the case of LLD are important from a clinical viewpoint. However, no clearly defined and widely accepted solution presenting an optimal treatment strategy has yet been reached. A discrepancy of less than 20 mm is a common phenomenon and, as a rule, does not lead to serious clinical consequences. On the other hand, a difference of 20 mm or more is thought to be clinically significant and usually requires adequate treatment to be implemented [17].

This study aimed to investigate the relationship between leg length discrepancy and spinal curves in the frontal plane in individuals with scoliotic posture and scoliosis. The study was designed to provide a better understanding of compensatory mechanisms and the posture-related factors associated with spinal deformities, as well as to support more accurate diagnosis and treatment planning.

The findings provide valuable insights into the relationship between scoliometer measurements and biomechanical as well as functional parameters. These findings may help optimise treatment strategies and serve as a starting point for further research into the treatment of patients with scoliosis.

## 2. Material and Methods

### 2.1. Sample Size Calculation

An *a priori* sample size estimation was performed using G*Power software (version 3.1.9.6, Düsseldorf, Germany). Based on an expected moderate correlation coefficient (*r* = 0.45 to 0.5) between clinical asymmetry indices and spinal parameters, with an alpha level of *α* = 0.05 and a statistical power (1 – *β*) of 0.80, a minimum required sample size of *n* = 29 participants was established.

**Figure 1:**
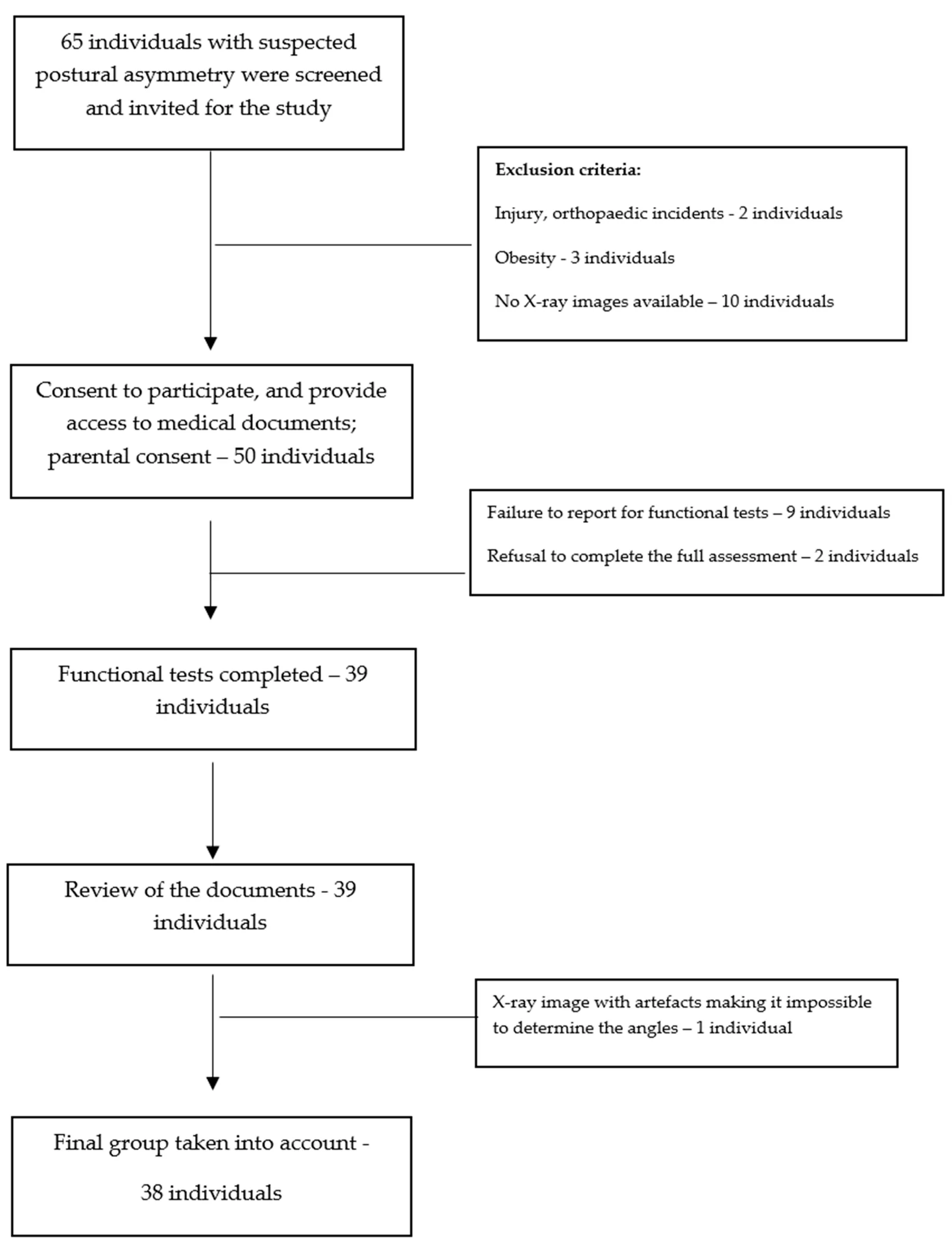
Flow of participants in the study.

Initially, a broader clinical cohort of *n* = 65 individuals with suspected postural asymmetries was invited for screening. However, after applying strict eligibility criteria, excluding individuals without the required full-spine X-rays or complete functional evaluations, and removing unreadable radiographic files, the analysed sample consisted of *n* = 38 participants (Figure 1). This sample size exceeds the prespecified minimum requirement of *n* = 29.

### 2.2. Participants

The study involved individuals aged 10 to 36 years with suspected postural disorders or musculoskeletal asymmetry, including pelvic misalignment, suspected LLD, or scoliosis, as reported by the patient or clinically identified.

The study took into account 38 individuals, on average aged 15.3 years (SD 5.78), from the city of Rzeszów, with a mean body weight of 55.3 kg (±12.66) and a mean height of 163.2 cm (±11.82) (Table 1).

The initial study population comprised individuals evaluated for suspected spinal or postural asymmetries. To ensure alignment with the scope presented in the Introduction, scoliosis was radiographically defined according to standard clinical criteria as a Cobb angle of ≥10° in the frontal plane accompanied by vertebral rotation.

Out of the 38 participants included in the final analysis (mean age 15.3 ± 5.78 years, range 10–36 years), 50% met the strict radiological threshold for confirmed scoliosis (Cobb angle ≥10°, while the remaining participants exhibited functional postural asymmetries/asymmetrical trunk rotation without structural spinal curvature. Furthermore, although the cohort included an age range of 10 to 36 years, over 75% of the participants *n = 29* were aged 13 to 16 years (IQR: 13.0–16.0 years), making the study population predominantly representative of an adolescent cohort presenting with either structural scoliosis or scoliotic posture.

Eligibility criteria included a referral or qualification for physiotherapeutic or orthopaedic evaluation due to postural asymmetry or scoliosis screening, availability of standing full-spine AP/lateral radiographs, the ability to stand and walk without assistance, and written consent to participate in the study and for the processing of anonymous data. Additionally, a referral for X-ray imaging issued by an orthopaedist or paediatrician was required.

The study protocol excluded individuals with existing injuries or those who had suffered injuries, accidents, or acute orthopaedic incidents within the six months prior to the study, as well as individuals who had undergone surgery on their lower limbs, pelvis, or spine. The following exclusion criteria were also applied: significant mobility limitations, severe obesity that impeded an accurate assessment of posture, pregnancy, and lack of consent to participate in the study or to the processing of personal data, as well as lack of the required referral for an X-ray examination. A detailed participant flow chart is shown in Figure 1.

### 2.3. Methods

The assessments were performed in the University Centre for Research and Development in Health Sciences at the University of Rzeszów in Poland and in Hospital No. 2 in the radiology laboratory. The examinations were carried out from April to October 2024. Both the participating participants and their parents were informed about the anonymity of the study, its subject matter, and its purpose; consent was obtained from the parents and individuals, the administration of all participating centres, and the bioethics committee (RESOLUTION No. 5/01/2024 of 17 January 2024, of the Physiotherapists’ Scientific Research Ethics Committee at the Polish Physiotherapy Association).

### 2.4. Physiotherapeutic Evaluation

A quantitative assessment of trunk asymmetry in the horizontal plane was performed using a Bunnell scoliometer (Orthopedic Systems Inc., Union City, CA, USA). The evaluation was performed using the forward bend test (Adam’s test). All clinical evaluations and scoliometer measurements were performed by a single, experienced physiotherapist to eliminate inter-examiner variability. The examiner moved the scoliometer along the spine, positioning the notch of the device over the spinous processes. Readings were taken at the points of greatest protrusion of the rib hump or the lumbar prominence [18,19,20,21].

To differentiate between true and apparent leg length discrepancy (the latter resulting from pelvic asymmetry), the Derbolovsky test (long-sitting test) was performed. The patient moved from a supine position to a long sitting position without using their hands. The examiner observed the change in the relative position of the medial malleoli. Any apparent difference in the length of the limbs during the transition to a sitting position was interpreted as a sign of pelvic rotation [22]. The selection of specific clinical tests (Derbolovsky, Standing Flexion, and Gillet tests) was guided by their targeted biomechanical mechanisms. The Derbolovsky test (long-sitting test) was used to differentiate functional (apparent) leg length discrepancy from anatomical asymmetry by evaluating relative functional shortening caused by pelvic/sacroiliac torsion during dynamic positional changes. The Standing Flexion Test was applied to assess symmetrical vs. asymmetrical mobility of the sacroiliac joints during global trunk flexion in a standing, weight-bearing posture. The Gillet test was selected to evaluate dynamic pelvic stability and sacroiliac joint locking during single-leg weight-bearing stance. Together, these tests allow for a comprehensive functional assessment of the pelvis–sacroiliac complex, helping to clarify whether lower limb discrepancies are fixed/anatomical or secondary to pelvic asymmetry. These palpatory manoeuvres were utilised as qualitative clinical indicators of pelvic asymmetry rather than for diagnosing structural pathology.

The mobility of the sacroiliac joints was assessed using the standing flexion test. The examiner, standing behind the patient, placed their thumbs on the posterior superior iliac spine (PSIS). The patient, standing with their knees fully extended, performed a slow bend forward with their trunk. The test was considered positive when one of the thumbs moved higher, suggesting limited mobility in the sacroiliac joint on that side [23,24].

The dynamic stability of the pelvis was assessed using the Gillet test. The examiner simultaneously palpated the PSIS on the tested side and the median sacral crest at the S2 level. The patient raised the leg, flexing the hip beyond 90°. Under physiological conditions, the iliac spine should move downward relative to the sacrum; the absence of this movement or its upward orientation was interpreted as joint locking [25].

Lower limb parameters included both relative length (measured clinically/radiographically from the anterior superior iliac spine to the medial malleolus) and absolute length (measured from the greater trochanter or femoral head to the medial malleolus/distal tibia). Calculated differences for both relative and absolute lengths were computed as left-to-right absolute delta values (|L−R|). Levelling shoe lift values represented the precise millimetre height of heel lifts currently used or clinically required to level the pelvis.

### 2.5. Radiological Assessment

The measurements were performed using the Exhibeon 2.40 software (Pixel Technology, Łódź, Poland) on frontal (AP) and lateral full-length spine radiographs, as well as frontal (AP) full-length radiographs of the lower extremities. All radiographic measurements (Cobb angle, Ferguson angle, sagittal curves, and LLD) were conducted by a single investigator to ensure methodological consistency. The distances and lengths, as well as the angles, were determined using the appropriate tools in this software. The angles were measured using a dedicated tool that determines the angle of intersection based on two lines drawn on the image—the Cobb method [26].

The length of the lower extremity was measured in a straight line from the tip of the femoral head to the midpoint of the articular surface of the distal tibial epiphysis at the ankle joint. To determine the difference in the height of the iliac bones, auxiliary horizontal lines were drawn on the X-rays—that is, lines parallel to the ground and tangent to both iliac crests; subsequently, the distance between these lines was determined by measuring the length of a perpendicular line intersecting both of them. The angles of the scoliotic deformities of the spine were measured in accordance with the Cobb method [26].

Axial vertebral rotation was assessed using the Nash–Moe scale, based on the projections of the vertebral pedicles relative to the vertebral body [27]. Angles of spinal curvatures in the sagittal plane were determined using the Cobb method [26]. Cervical lordosis was measured using the Cobb method from C2 to C7 on sagittal radiographs where the cervical spine was fully visible (n=27 out of 38 participants; in 11 cases, the cervical spine was outside the radiographic field of view).

Although the primary focus of this study was spinal deformity in the frontal plane, additional sagittal radiographic parameters (cervical lordosis, thoracic kyphosis, lumbar lordosis, Ferguson’s angle) and sacroiliac joint (SIJ) clinical mobility tests (Derbolovsky, Standing Flexion, Gillet) were included to evaluate potential multi-planar compensatory adaptations. This exploratory approach allowed us to assess whether localised frontal pelvic asymmetry triggers ascending kinematic chain responses extending to the cervical region.

### 2.6. Statistical Analysis

The entire empirical material collected was subjected to a statistical analysis performed using Statistica 13.3 (TIBCO Software Inc., Palo Alto, CA, USA).

In order to characterise the study group and the quantitative variables analysed, the measures of central tendency and dispersion were calculated: the arithmetic mean, standard deviation, median, and quartiles (Q1—the first quartile; Q3—the third quartile).

The strength and direction of the association between two quantitative variables were assessed using Spearman’s rank correlation coefficient to evaluate monotonic relationships (not necessarily linear), particularly when the assumptions of Pearson’s correlation were not met (e.g., small sample size or lack of normal distribution).

The normality of the distribution of quantitative variables within the analysed groups was assessed using the Shapiro–Wilk test.

For comparisons of quantitative variables between two independent groups, Student’s *t*-test for independent samples was used when the assumptions of parametric testing were met. Homogeneity of variances was assessed using Levene’s test; when this assumption was violated, Welch’s *t*-test was applied. When the distributional assumptions were not met, the non-parametric Mann–Whitney *U* test was used.

Categories comprising fewer than three observations were not included in inferential analyses. For the Derbolovsky test, the “No deformities” category, which included one or two participants depending on the availability of the analysed parameter, was retained for descriptive purposes only. Comparative analyses for this test were therefore performed between the groups with positive results on the right and left sides.

To reduce the risk of Type I error associated with multiple testing, the Bonferroni correction was applied within separate families of hypotheses defined according to the analysed outcome variable. For the analyses corresponding to Table 2, two correlations were included in each family separately for the scoliometer measurement and for the Cobb angle. For the analyses corresponding to Table 3, eight correlations were included in each family separately for the scoliometer measurement and for the Cobb angle. For the comparisons corresponding to Tables 4–9, three clinical-test comparisons were included in each family for each analysed outcome variable. The same family definitions and correction procedure were retained in the age-restricted sensitivity analyses. Adjusted probability values were denoted as *p_adj_*.

To assess the robustness of the findings with regard to the wide age range of the study sample, a sensitivity analysis was performed after restricting the dataset to participants younger than 18 years. The principal correlation analyses and comparisons based on the clinical tests were repeated in this age-restricted subgroup using the same statistical procedures and the same Bonferroni correction scheme as in the primary analysis. Additionally, an exploratory subgroup analysis was performed among participants with radiographically confirmed structural scoliosis, defined as a Cobb angle ≥10°. Within this subgroup, Spearman’s rank correlations between the angle of trunk rotation (ATR) and the radiographic indicators of lower limb and pelvic asymmetry were recalculated. Due to the reduced subgroup sample size, these analyses were interpreted cautiously.

For all statistical analyses, the significance level was set at α = 0.05. For analyses subject to correction for multiple comparisons, results were considered statistically significant when the adjusted *p_adj_* value was below 0.05.

## 3. Results

### 3.1. Relationship Between Lower Limb Asymmetry and Frontal Spinal Deformity (ATR vs. Cobb Angle)

Trunk rotation (scoliometer measurement for the primary curve) demonstrated moderate, positive correlations with both lower limb asymmetry indicators: leg length discrepancy *(r_s_* = 0.40, *p_adj_* = 0.041) and pelvic height difference *(r_s_* = 0.40, *p_adj_* = 0.040). Both associations retained statistical significance following Bonferroni correction, whereas no significant correlations were observed with the primary curve Cobb angle (Table 2).

Trunk rotation (scoliometer measurement) demonstrated statistically significant positive correlations with both relative *(r_s_* = 0.45, *p_adj_* = 0.043) and absolute *(r_s_* = 0.53, *p_adj_* = 0.005) lower limb length differences, retaining significance after Bonferroni correction. No other limb length parameters or corrective insole values showed significant associations with trunk rotation.

Similarly, none of the evaluated functional asymmetry and shoe lift parameters exhibited statistically significant correlations with radiological Cobb angle measurements following the Bonferroni adjustment (Table 3).

In the sensitivity analysis (*n* = 35, age < 18), correlations between trunk rotation (ATR) and primary radiographic indicators attenuated slightly, becoming non-significant after Bonferroni correction for both LLD *(r_s_* = 0.34, *p_adj_* = 0.114) and pelvic height difference *(r_s_* = 0.33, *p_adj_* = 0.128). However, the association between ATR and absolute leg-length difference remained robust and statistically significant *(r_s_* = 0.50, *p_adj_* = 0.021). Relationships between lower limb indicators and Cobb angle remained consistently non-significant *(p_adj_* = 1.000).

In the structural scoliosis subgroup (Cobb ≥ 10°, *n* = 19), ATR maintained positive correlation trends with LLD *(r_s_* = 0.36, *p_adj_* = 0.271) and pelvic height difference *(r_s_* = 0.35, *p_adj_* = 0.279), though these did not reach statistical significance following Bonferroni adjustment.

### 3.2. Impact of Clinical Sacroiliac Dysfunction Tests on Frontal and Sagittal Alignment

Across all three clinical assessments (Derbolovsky, standing flexion, and Gillet tests), no statistically significant group differences were observed for trunk rotation (ATR) or any radiological parameter (Cobb angle, thoracic kyphosis, lumbar lordosis, and Ferguson angle), both in the total cohort and the under-18 sensitivity analysis following Bonferroni correction (Table 4, Table 5, Table 6, Table 7 and Table 8). Due to insufficient group size *n* ≤ 2, the “no deformities” category of the Derbolovsky test was excluded from inferential testing and evaluated descriptively, with group comparisons performed between right- and left-sided positives.

### 3.3. Exploratory Analysis of Ascending Compensations: Clinical Tests vs. Cervical Lordosis

In contrast, cervical lordosis differed significantly based on clinical test classification (Table 9). Participants with positive left-sided test results exhibited significantly higher cervical lordosis values than those with right-sided positive results across all three clinical assessments: Derbolovsky (*p_adj_* = 0.025), standing flexion (*p_adj_* < 0.001), and Gillet tests (*p_adj_* < 0.001).

This pattern remained fully consistent in the <18 sensitivity analysis (*n* = 24) with significantly greater cervical lordosis in left-positive participants across the Derbolovsky (*p_adj_* = 0.004), standing flexion (*p_adj_* = 0.009), and Gillet tests (*p_adj_* = 0.009) following Bonferroni correction.

The original source data were rechecked, explicitly confirming that within the subgroups with available cervical lordosis measurements, the Standing Flexion and Gillet tests yielded identical right/left classifications; therefore, the corresponding descriptive and inferential results are identical.

## 4. Discussion

The present findings may suggest that leg length discrepancy (LLD) is prevalent among patients with scoliosis, being significantly associated with pelvic asymmetry and alterations in spinal alignment within the frontal plane. These results are consistent with previous studies showing that a significant proportion of patients with scoliosis exhibit measurable LLD. Failure to account for this may lead to misinterpretation of spinal deformity [28]. D’Amico et al. pointed out that patients with scoliosis typically present with an LLD of approximately 10 mm on average, and that correcting lower limb asymmetry may lead to improved postural parameters and a reduction in the degree of spinal curvature. This highlights the importance of this factor in diagnosis and conservative treatment [28].

The need to distinguish between structural and functional leg length discrepancy (LLD) is increasingly emphasised in the literature. Studies using advanced imaging techniques, including low-dose EOS systems, have revealed that functional LLD is more closely linked to pelvic obliquity and the Cobb angle in the lumbar spine. In contrast, structural LLD tends to be smaller and less correlated with spinal deformity. These findings suggest that biomechanical compensatory mechanisms rather than anatomical differences play a key role in the development of scoliosis, particularly the functional type [29].

Many radiographic studies have also confirmed correlations between LLD and spinal curvature. Hamada et al. demonstrated a positive relationship between the absolute difference in leg length, expressed in millimetres, the relative leg-length discrepancy ratio (LLDR), and the Cobb angle in the lumbar spine. This indicates that greater leg length asymmetries are associated with more severe spinal deformity [30]. Importantly, this correlation was stronger for relative ratios than for absolute values, confirming the clinical relevance of taking leg-length ratios into account when assessing posture biomechanics.

The biomechanical basis of the observed relationships has been extensively described in the literature. LLD is strongly correlated with pelvic obliquity in the frontal plane, which usually occurs alongside lateral curvature of the spine, most frequently in the lumbar region, with the curve’s convexity oriented towards the shorter limb [13]. This mechanism is particularly evident in children, whose musculoskeletal systems are highly malleable. Studies have shown that using shoe inserts to correct LLD can significantly reduce scoliotic deviations in the body’s axes, particularly in cases of functional scoliosis [6,13,28].

While LLD values reported in the literature vary depending on population and measurement method, most authors emphasise that even subtle differences in leg length, not exceeding 10 mm, can be associated with significant changes in pelvic and spinal biomechanics. From a clinical viewpoint, such differences are particularly important during periods of rapid growth, as compensatory mechanisms can reinforce abnormal postural patterns [3,7].

The results obtained suggest the usefulness of the scoliometer as a screening tool for assessing scoliosis. There are significant correlations between angle of trunk rotation (ATR) values and radiological parameters, including the Cobb angle and degree of pelvic asymmetry [13,31]. At the same time, numerous studies indicate that ATR measurements are sensitive to biomechanical factors such as LLD (limb length discrepancy) and pelvic obliquity. These factors may be linked to over- or underestimation of results in clinical trials [32,33,34].

Studies in which simulated changes in pelvic position ranging from 1 to 2 cm were associated with significant shifts in ATR values in various spinal segments, particularly in the lumbar region, have supported the relationship between pelvic asymmetry and ATR values. These results may suggest that an assessment of pelvic alignment and possible LLD should always be included when interpreting scoliometer measurements to avoid incorrect classification of patients in screening tests [35].

Radiographic examinations (X-rays) enable an objective assessment of the effect of shoe inserts on the alignment of the pelvis and spine. D’Amico et al. demonstrated that the use of corrective insoles led to a visible reduction in pelvic asymmetry in the anteroposterior projection, and in patients with functional scoliosis a simultaneous decrease in the Cobb angle in the lumbar spine was observed [28]. Similar results were reported by Raczkowski et al., who confirmed that the use of corrective inserts to compensate for LLD resulted in the normalisation of pelvic obliquity and a reduction in the lateralisation of the trunk axis of the body [13].

In contrast to several published studies that reported significant correlations between LLD and the radiographic Cobb angle (particularly in lumbar curves), our study identified no statistically significant correlation between LLD indicators and the Cobb angle. This discrepancy can be attributed to several methodological and cohort characteristics. First, our cohort comprised predominantly mild-to-moderate curves, whereas studies demonstrating strong LLD–Cobb correlations often evaluate cohorts with larger structural lumbar curves. Second, LLD in our sample was generally mild (mean < 10 mm). In such ranges, pelvic tilt can be buffered by localised lumbar flexibility without escalating the primary structural Cobb angle. Crucially, while LLD did not track structural curve magnitude (Cobb angle), it significantly correlated with trunk rotation (ATR). This suggests that lower limb asymmetry primarily is associated with soft-tissue pelvic tilting and apparent surface trunk rotation without necessarily driving structural vertebral remodelling in mild curvature spectrums.

Furthermore, regarding clinical management and the consideration of orthotic interventions—such as shoe lifts or heel wedges for functional LLD—particular caution is warranted. Because our radiographic protocol measured LLD down to the distal tibia at the ankle joint without evaluating foot arch height, pes planus, or talar tilt, standing pelvic obliquity in some patients may be partially driven or amplified by asymmetric foot posture. Consequently, a detailed clinical and podiatric assessment of foot architecture must precede any prescription of compensatory shoe lifts to avoid overcorrecting functional adaptations.

A key and unexpected finding of this study was the pronounced difference in cervical lordosis based on the laterality of clinical indicators of pelvic asymmetry. Participants with positive findings on the left side (in Derbolovsky, Standing Flexion, and Gillet tests) exhibited substantially greater cervical lordosis angles compared to those with right-sided positives. Biomechanically, this observation raises the hypothesis of an ascending postural interaction where asymmetrical pelvic torsion alters the spatial orientation of the sacral base (Ferguson’s angle), inducing compensatory adjustments in the spine. To maintain horizontal gaze alignment and postural equilibrium, the cervical spine may execute counter-balancing sagittal adjustments, potentially amplified by asymmetric paraspinal muscle tone [36,37,38]. However, given the reduced sample size for cervical radiographs (*n* = 27), the dynamic variability of unstandardised head positioning during X-rays, and the cross-sectional design of our study, this intriguing biomechanical finding must be considered strictly hypothesis-generating rather than proof of a fixed closed ascending chain. To formally validate this ascending compensatory model, future longitudinal research should utilize full-spine 3D biplanar imaging (e.g., EOS system), surface electromyography (sEMG) of paraspinal musculature, and finite element modelling to isolate segment-by-segment spinal shifts driven by pelvic asymmetry [39].

## 5. Limitations

Several limitations of the present study should be acknowledged.

First, the cross-sectional and observational design precludes any conclusions regarding causality or the temporal sequence of events. While our findings demonstrate significant correlations between lower limb discrepancy (LLD) and the angle of trunk rotation (ATR), we cannot determine whether LLD initiates compensatory spinal adjustments or if primary scoliotic deformities contribute to secondary asymmetrical weight-bearing and functional LLD.

Second, the clinical tests used to assess sacroiliac joint mobility (Derbolovsky, Standing Flexion, and Gillet tests) carry inherent palpatory subjective limitations. Intra-rater diagnostic reliability (e.g., Cohen’s kappa) was not prospectively calculated for this specific trial. Therefore, positive test results should be interpreted as palpatory clinical indicators of pelvic asymmetry rather than definitive structural pathology.

Third, the methodological scope of the LLD measurement was limited to radiographic parameters from the femoral heads to the distal epiphysis of the tibia at the ankle joint. This approach does not account for foot morphology, such as foot arch height, pes planus (flatfoot), or talar tilt, which can introduce several millimetres of apparent functional asymmetry in a standing posture. Consequently, foot-driven contributions to pelvic obliquity could not be fully isolated, which is particularly relevant when evaluating shoe lift indications.

Fourth, the study lacked healthy controls or specific comparison groups (e.g., individuals with LLD without scoliosis or scoliosis patients without LLD). As all 38 participants were recruited from a single clinical centre, selection bias cannot be ruled out, and the findings should be interpreted specifically within this mixed cohort rather than generalised broadly to the entire population. Additionally, cervical lordosis could only be evaluated in a subset of participants *n* = 27 due to radiographic field-of-view limits. Furthermore, standing cervical posture on sagittal X-rays depends heavily on dynamic head positioning, visual gaze, and positioning instructions, which were not standardised using fixed optical targets. Consequently, these sagittal findings must be treated strictly as preliminary and exploratory.

Finally, the relatively small sample size and wide age distribution (10–36 years) introduce potential heterogeneity regarding skeletal maturity and spinal flexibility. Although the age range spanned from 10 to 36 years, over 75% of the study cohort comprised individuals aged 13 to 16 years (mean age 15.3 years). Future studies employing longitudinal designs, strict age-stratified cohorts, foot biomechanics assessment, and control groups are recommended to further elucidate these biomechanical mechanisms.

## 6. Conclusions

Scoliometer measurements reflect surface trunk rotation and are sensitive to lower extremity and pelvic asymmetries, yet they show no direct association with the structural severity of spinal curvature (radiographic Cobb angle).

While whole-cohort analyses suggested moderate correlations between ATR and primary radiographic LLD/pelvic height difference, these associations attenuated and lost statistical significance in both age-restricted (<18 years, *n* = 35) and structural scoliosis (Cobb ≥ 10°, *n* = 19) sensitivity analyses. Crucially, only the relationship between ATR and absolute leg-length difference remained robust and statistically significant in the adolescent population *(r_s_* = 0.50, *p_adj_* = 0.021).

These findings highlight that surface trunk asymmetry in scoliotic posture is influenced by postural leg length factors, but these relationships must be interpreted with caution across heterogeneous age and curve spectrums. Furthermore, while standard palpatory clinical tests (Derbolovsky, standing flexion, and Gillet) did not differentiate frontal plane scoliosis severity or ATR, their observed relationship with cervical lordosis suggests preliminary, exploratory sagittal compensations that warrant future multi-planar 3D evaluation.

## Figures and Tables

**Table 1:** Descriptive statistics—age, body height, and body weight.

Variables	Descriptive Statistics							
	*n*	$\bar{x}$	Me	Min.	Max.	Q1	Q3	*SD*
**Age [years]**	38	15.3	14.0	10.0	36.0	13.0	16.0	5.78
**Body height [cm]**	38	163.2	163.0	140.0	191.0	156.0	172.0	11.82
**Body weight [kg]**	38	55.3	53.5	29.0	83.0	45.0	65.0	12.66

N—Number of observations; $\bar{x}$—mean; Me—median; Min.—minimum value; Max.—maximum value; Q1—lower quartile; Q3—upper quartile; *SD*—standard deviation.

**Table 2:** Spearman’s correlation between asymmetry indicators (X-ray) and scoliometer measurements (First arc) and the Cobb Angle (First arc).

Variables	Scoliometer—First Arc			Cobb Angle—First Arc		
	*r*_s_	*p*	*p*_adj_	*r*_s_	*p*	*p*_adj_
**Difference in leg length**	**0.40**	**0.021**	**0.041**	0.05	0.761	1.000
**Difference in the height of the iliac bones**	**0.40**	**0.020**	**0.040**	0.02	0.894	1.000

*r*_s_—Spearman’s rank correlation coefficient; *p*—probability value; *p*_adj_—*p*-value adjusted using the Bonferroni correction; Note: statistically significant results have been bolded

**Table 3:** Correlations between leg length and shoe lift indices versus scoliometer parameters and the Cobb Angle.

Variables	Scoliometer—First Arc			Cobb Angle—First Arc		
	*r*_s_	*p*	*p*_adj_	*r*_s_	*p*	*p*_adj_
**Relative length L**	0.02	0.897	1.000	−0.03	0.869	1.000
**Relative length R**	0.07	0.693	1.000	−0.03	0.842	1.000
**Absolute length L**	0.04	0.835	1.000	0.00	0.987	1.000
**Absolute length R**	0.08	0.629	1.000	0.01	0.968	1.000
**Levelling shoe lift L**	0.26	0.138	1.000	0.04	0.812	1.000
**Levelling shoe lift R**	0.05	0.797	1.000	0.07	0.697	1.000
**Relative length—difference**	**0.45**	**0.005**	**0.043**	0.09	0.625	1.000
**Absolute length—difference**	**0.53**	**0.001**	**0.005**	0.08	0.631	1.000

*r*_s_—Spearman’s rank correlation coefficient; *p*—probability value; *p*_adj_—*p*-value adjusted using the Bonferroni correction; Note: statistically significant results have been bolded

**Table 4:** Comparison of scoliometer (first arc) values in the groups distinguished based on the clinical tests.

Variables		Scoliometer (First Arc)								
		N	$\bar{x}$	Me	Min.	Max.	Q1	Q3	*SD*	*p*
**Derbolovsky test**	**Right**	13	5.77	4.0	1.0	18.0	3.0	6.0	4.66	Z = −0.20 0.840 *p_adj_* = 1.000
	**Left**	23	4.26	4.0	1.0	8.0	3.0	5.0	1.71	
**Standing flexion test**	**Right**	11	6.09	4.0	1.0	18.0	3.0	10.0	5.03	Z = −0.45 0.654 *p_adj_* = 1.000
	**Left**	26	4.19	4.0	1.0	8.0	3.0	5.0	1.63	
**Gillet test**	**Right**	12	5.83	4.0	1.0	18.0	3.0	8.0	4.88	Z = −0.10 0.923 *p_adj_* = 1.000
	**Left**	25	4.24	4.0	1.0	8.0	4.0	5.0	1.64	

N—Number of observations; $\bar{x}$—mean; Me—median; Min.—minimum value; Max.—maximum value; Q1—lower quartile; Q3—upper quartile; *SD*—standard deviation; Z—value of Mann–Whitney U-test statistic; *p*—probability value; *p_adj_*—*p*-value adjusted using the Bonferroni correction. *n* = 37 due to missing clinical scoliometer (ATR) data for one participant.

**Table 5:** Comparison of the X-ray Cobb Angle values in the groups distinguished based on the clinical tests.

Variables		X-Ray (Cobb Angle)								
		N	$\bar{x}$	Me	Min.	Max.	Q1	Q3	*SD*	*p*
**Derbolovsky test**	**Right**	12	9.99	7.5	0.7	26.0	5.0	14.0	7.31	Z = 1.28 0.199 *p_adj_* = 0.598
	**Left**	22	11.77	12.0	0.4	26.0	8.0	14.4	5.70	
	**No deformities**	1	8.30	8.3	8.3	8.3	8.3	8.3	-	
**Standing flexion test**	**Right**	10	10.19	6.6	0.7	26.0	5.0	14.0	8.05	Z = 1.10 0.273 *p_adj_* = 0.819
	**Left**	25	11.41	11.0	0.4	26.0	8.0	12.0	5.43	
**Gillet test**	**Right**	11	10.02	6.9	0.7	26.0	5.0	14.0	7.66	Z = 1.21 0.227 *p_adj_* = 0.681
	**Left**	24	11.54	11.5	0.4	26.0	8.0	13.2	5.51	

N—Number of observations; $\bar{x}$—mean; Me—median; Min.—minimum value; Max.—maximum value; Q1—lower quartile; Q3—upper quartile; *SD*—standard deviation; Z—value of Mann–Whitney U-test statistic; *p*—probability value; *p_adj_*—*p*-value adjusted using the Bonferroni correction. *n* = 35 due to missing or unreadable full-spine radiographs in three participants.

**Table 6:** Comparison of the X-ray Th kyphosis in the groups distinguished based on the clinical tests.

Variables		X-Ray (Th Kyphosis)								
		N	$\bar{x}$	Me	Min.	Max.	Q1	Q3	*SD*	*p*
**Derbolovsky test**	**Right**	9	36.53	38.0	17.0	63.0	22.0	47.0	16.03	t = −0.40 0.696 *p_adj_* = 1.000
	**Left**	17	34.24	35.0	1.0	52.0	27.0	43.0	13.05	
	**No deformities**	2	23.50	23.5	20.0	27.0	20.0	27.0	4.95	
**Standing flexion test**	**Right**	7	36.69	38.0	17.0	63.0	21.0	50.8	17.38	t = −0.54 0.591 *p_adj_* = 1.000
	**Left**	21	33.38	35.0	1.0	52.0	25.0	43.0	12.67	
**Gillet test**	**Right**	8	34.60	30.0	17.0	63.0	20.5	47.9	17.14	t = −0.09 0.926 *p_adj_* = 1.000
	**Left**	20	34.05	35.0	1.0	52.0	26.0	44.0	12.61	

N—Number of observations; $\bar{x}$—mean; Me—median; Min.—minimum value; Max.—maximum value; Q1—lower quartile; Q3—upper quartile; *SD*—standard deviation; t—value of Student’s *t*-test statistic for independent samples; *p*—probability value; *p_adj_*—*p*-value adjusted using the Bonferroni correction. *n* = 28 because thoracic kyphosis could not be determined on sagittal radiographs due to poor visibility or field-of-view limitations.

**Table 7:** Comparison of the X-ray L lordosis in the groups distinguished based on the clinical tests.

Variables		X-Ray (L lordosis)								
		N	$\bar{x}$	Me	Min.	Max.	Q1	Q3	*SD*	*p*
**Derbolovsky test**	**Right**	10	44.90	44.0	30.0	62.0	37.0	48.0	10.16	t = −0.74 0.469 *p_adj_* = 1.000
	**Left**	16	42.26	43.0	25.0	55.0	39.1	47.5	8.02	
	**No deformities**	2	53.00	53.0	46.0	60.0	46.0	60.0	9.90	
**Standing flexion test**	**Right**	8	48.00	46.0	37.0	62.0	42.5	54.0	8.72	t = −1.53 0.139 *p_adj_* = 0.417
	**Left**	20	42.36	43.0	25.0	60.0	37.0	47.5	8.88	
**Gillet test**	**Right**	9	47.78	46.0	37.0	62.0	43.0	48.0	8.18	t = −1.57 0.128 *p_adj_* = 0.383
	**Left**	19	42.17	42.0	25.0	60.0	35.0	48.0	9.08	

N—Number of observations; $\bar{x}$—mean; Me—median; Min.—minimum value; Max.—maximum value; Q1—lower quartile; Q3—upper quartile; *SD*—standard deviation; t—value of Student’s *t*-test statistic for independent samples; *p*—probability value; *p_adj_*—*p*-value adjusted using the Bonferroni correction. *n* = 28 because lumbar lordosis could not be determined on sagittal radiographs due to poor visibility or field-of-view limitations.

**Table 8:** Comparison of the X-ray Ferguson’s Angle values in the groups distinguished based on the clinical tests.

Variables		X-Ray (Ferguson’s Angle)								
		N	$\bar{x}$	Me	Min.	Max.	Q1	Q3	*SD*	*p*
**Derbolovsky test**	**Right**	10	35.53	35.2	28.0	47.0	30.0	39.0	6.14	t = 0.60 0.553 *p_adj_* = 1.000
	**Left**	16	36.79	37.0	26.0	45.0	36.0	39.5	4.51	
	**No deformities**	2	33.25	33.3	24.5	42.0	24.5	42.0	12.37	
**Standing flexion test**	**Right**	8	36.54	37.7	28.0	47.0	30.5	40.5	6.48	Z = −0.33 0.741 *p_adj_* = 1.000
	**Left**	20	35.91	37.0	24.5	45.0	32.5	39.5	5.27	
**Gillet test**	**Right**	9	35.20	37.3	24.5	47.0	30.0	39.0	7.27	t = 0.58 0.569 *p_adj_* = 1.000
	**Left**	19	36.51	37.0	26.0	45.0	33.0	40.0	4.66	

N—Number of observations; $\bar{x}$—mean; Me—median; Min.—minimum value; Max.—maximum value; Q1—lower quartile; Q3—upper quartile; *SD*—standard deviation; t—value of Student’s *t*-test statistic for independent samples; Z—value of Mann–Whitney U-test statistic; *p*—probability value; *p*_adj_—*p*-value adjusted using the Bonferroni correction; n = 28 because Ferguson’s Angle could not be determined on sagittal radiographs due to poor visibility or field-of-view limitations.

**Table 9:** Comparison of the X-ray C lordosis in the groups distinguished based on the clinical tests (Derbolovsky test, Standing flexion test, Gillet test).

Variables		X-Ray (C Lordosis)								
		N	$\bar{x}$	Me	Min.	Max.	Q1	Q3	*SD*	*p*
**Derbolovsky test**	**Right**	10	11.03	8.7	2.0	35.0	6.0	14.0	9.75	**Z = −2.64** **0.008** ***p_adj_*** ** = 0.025**
	**Left**	16	26.85	25.0	5.0	51.0	16.5	39.0	14.86	
	**No deformities**	1	18.00	18.0	18.0	18.0	18.0	18.0	-	
**Standing flexion test**	**Right**	8	9.16	8.7	2.0	17.0	6.0	12.5	4.94	t* = −4.24 **<0.001** ***p_adj_*** ** = <0.001**
	**Left**	19	25.51	23.0	2.0	51.0	14.0	38.0	14.98	
**Gillet test**	**Right**	8	9.16	8.7	2.0	17.0	6.0	12.5	4.94	t* = −4.24 **<0.001** ***p_adj_*** ** = <0.001**
	**Left**	19	25.51	23.0	2.0	51.0	14.0	38.0	14.98	

N—Number of observations; $\bar{x}$—mean; Me—median; Min.—minimum value; Max.—maximum value; Q1—lower quartile; Q3—upper quartile; *SD*—standard deviation; t*—value of Welch’s *t*-test statistic; Z—value of Mann–Whitney U-test statistic; *p*—probability value; *p_adj_*—*p*-value adjusted using the Bonferroni correction. Note: Cervical lordosis measurements were available for a subset of *n* = 27 participants due to field of view limitations on sagittal standing X-rays; Note: statistically significant results have been bolded.

## Data Availability

All data are available from the corresponding author.

## Abbreviations

The following abbreviations are used in this manuscript:

AIS	adolescent idiopathic scoliosis
PA	posteroanterior
LLD	leg length discrepancy
SD	standard deviation
Q1	first quartile
Q3	third quartile
N	number of observations
X	average value
Me	median
Min	minimum value
Max	maximum value
PSIS	posterior superior iliac spine
AP	anterior–posterior
ATR	trunk rotation angle
